# Diagnostic value of CT enhancement degree in lymph node metastasis of papillary thyroid cancer: A comparison of enhancement, ratio, and difference

**DOI:** 10.3389/fendo.2023.1103434

**Published:** 2023-03-22

**Authors:** Jiying Zhu, Min Tian, Tong Zhang, Hanlin Zhu, Peiying Wei, Zhijiang Han

**Affiliations:** ^1^ Department of Radiology, Affiliated Hangzhou First People’s Hospital, Zhejiang University School of Medicine, Hangzhou, China; ^2^ Department of Radiology, Hangzhou Ninth People’s Hospital, Hangzhou, China

**Keywords:** lymph node metastasis, thyroid nodule, thyroid tumor, tomography, x-ray computer

## Abstract

**Objectives:**

To evaluate the value of computed tomography (CT) enhancement degree in diagnosing lymph node (LN) metastasis in papillary thyroid carcinoma (PTC) by determining the ratio and difference between the Hounsfield units (HU) of CT enhancement and plain scan of the LNs, as well as between the HU of CT-enhanced LNs and the sternocleidomastoid muscle.

**Methods:**

The plain and enhanced CT findings of 114 metastasis-positive LNs in 89 cases and 143 metastasis-negative LNs in 114 cases of PTC were analyzed retrospectively. Plain HU of LNs (PN_HU_), enhanced HU of LNs (EN_HU_), and enhanced HU of the sternocleidomastoid muscle (EM_HU_) were measured. The EN_HU_, difference between EN_HU_ and PN_HU_ (EN-PN_HU_), ratio of EN_HU_ to PN_HU_ (EN/PN_HU_), difference between EN_HU_ and EM_HU_ (EN-EM_HU_), and ratio of EN_HU_ to EM_HU_ (EN/EM_HU_) in metastasis-positive and metastasis-negative LN groups were calculated, the corresponding diagnostic efficacy for differentiating metastasis-positive from metastasis-negative LNs in PTC were sought using the receiver-operating curve. The interobserver agreement between readers was assessed using the interobserver correlation coefficient (ICC).

**Results:**

The EN_HU_ of 114 metastasis-positive LNs and 143 metastasis-negative LNs was 113.39 ± 24.13 and 77.65 ± 15.93, EN-PN_HU_ was 65.84 ± 21.72 HU and 34.07 ± 13.63 HU, EN/PN_HU_ was 2.36 (1.98, 2.75) and 1.76 (1.54, 2.02), EN-EM_HU_ was 49.42 ± 24.59 HU and 13.27 ± 15.41 HU, and EN/EM_HU_ was 1.79 ± 0.40 and 1.21 ± 0.24, respectively (all *P* < 0.001). The area under the curve, cutoff value, sensitivity, specificity, and accuracy of EN_HU_ for identifying metastasis-positive and metastasis-negative LNs were 0.895, 97.3 HU, 0.746, 0.895, and 0.829, EN-PN_HU_ was 0.894, 47.8 HU, 0.807, 0.874, and 0.844, EN/PN_HU_ was 0.831, 1.9, 0.877, 0.650, and 0.751, EN-EM_HU_ was 0.890, 26.4 HU, 0.807, 0.839, and 0.825, and EN/EM_HU_ was 0.888, 1.5, 0.728, 0.902, and 0.825, respectively. The readers had an excellent interobserver agreement on these five parameters (ICC = 0.874–0.994).

**Conclusion:**

In the preoperative evaluation of LN metastasis in PTC, EN_HU_, EN-PN_HU_, EN-EM_HU_, and EN/EM_HU_ had similarly high diagnostic efficacy, with EN_HU_, EN-PN_HU_, and EN/EM_HU_ having higher specificity and EN-PN_HU_ and EN-EM_HU_ having higher sensitivity.

## 1 Introduction

Papillary thyroid carcinoma (PTC) is the most common malignant tumor of the thyroid, accounting for 83.6%–88% of cases (1, 2). Although the 5-year and 10-year survival rates of low-risk PTC are nearly 100% (3, 4), 20%–90% of patients with PTC have cervical lymph node (LN) metastasis at the time of diagnosis (5), especially central LN metastasis. Metastatic LNs can invade the peripheral blood vessels, trachea, and esophagus, causing the stenosis of the corresponding lumen, and invade the recurrent laryngeal nerve, causing hoarseness. Prophylactic LN dissection can reduce the residual metastatic LNs, but it is bound to increase the risk of the parathyroid gland and recurrent laryngeal nerve injury (6). Therefore, accurately identifying and prophylactically removing the central metastasis-positive LN or high-risk groups at risk of LN metastasis while removing the primary tumor of PTC is important.

Ultrasonography is the most commonly used imaging modality during the preoperative evaluation and monitoring of cervical LN metastasis in PTC. Typical ultrasound (US) images of LN metastasis often show signs such as LNs with transverse/long diameter > 0.5, blurred corticomedullary demarcation, disappearance of medullary structures, microcalcifications, and cystic changes (7, 8). While assessing LN metastasis in the lateral cervical levels, the sensitivity and accuracy of ultrasonography diagnosis ranged 64%–74.3% and 70.0%–89.2%, respectively (9–13). However, in the assessment of LNs in the central levels, ultrasonography was often interfered with by gases in the trachea and esophagus, as well as occlusion of the sternum and clavicle; also, the efficacy of its assessment was not satisfactory, with the sensitivity and accuracy of 17.3%–38% and 58.5%–71%, respectively (9, 11–13). Compared with ultrasonography, computed tomography (CT) examination is not limited by gas and bone and can better show the central level LNs. However, micrometastases in the cervical LNs of PTC are more common, and most of these LNs do not have typical CT metastasis signs such as short diameter >1 cm, central necrosis or cystic degeneration, and microcalcifications. Besides, no accurate quantitative value is available for judging LN metastasis based on the criterion of “the degree of enhancement is higher than that of muscle as a suspicious LN.” Therefore, the sensitivity and accuracy of the CT examination are not superior to those of US, with 23.5%–50% and 55.7%–75%, respectively (11–13).

In the present study, we propose for the first time a comparative study of the LNs in one cervical level confirmed to be all metastasis-positive or all metastasis-negative, and the metastasis-positive LNs determined by fine-needle aspiration cytology (FNAC) under US-CT fusion navigation. The plain Hounsfield unit of LNs (PN_HU_), enhanced Hounsfield unit of lymph nodes (EN_HU_), enhanced Hounsfield unit of the sternocleidomastoid muscle (EM_HU_), difference between EN_HU_ and PN_HU_ (EN – PN_HU_), ratio of EN_HU_ to PN_HU_ (EN/PN_HU_), difference between EN_HU_ and EM_HU_ (EN – EM_HU_),and ratio of EN_HU_ to EM_HU_ (EN/EM_HU_) were measured, aiming to find the optimal threshold for differentiating the metastatic LNs and provide an important basis for clinicians to individualize treatment options.

## 2 Materials and methods

### 2.1 Participants

**Figure 1:**
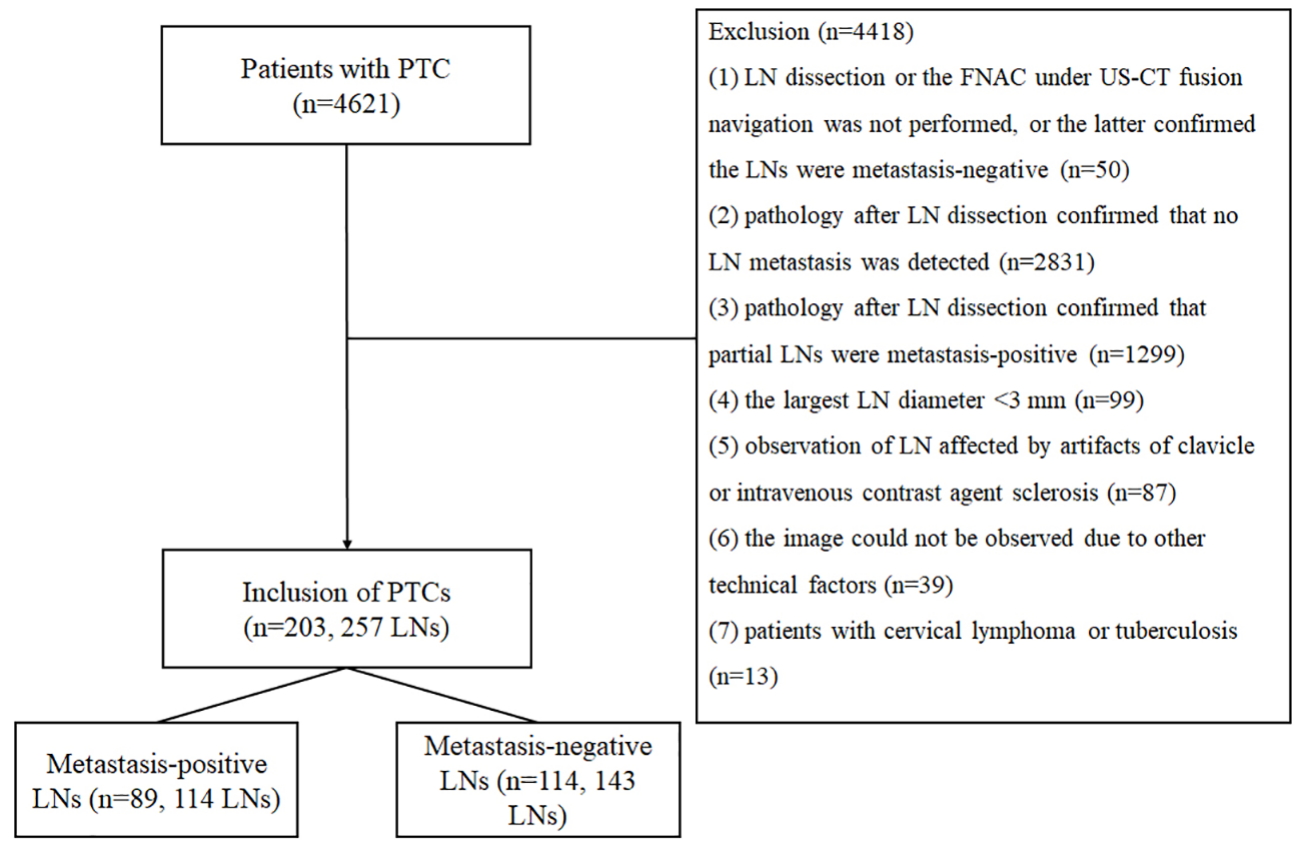
Flow chart of the study participants.

The study was performed following the ethical guidelines of the Helsinki Declaration. The ethics committee of Affiliated Hangzhou First People’s Hospital (IRB-2020-154) approved the study. Written informed consent for participation was waived due to the retrospective nature of the study and the use of anonymized patient data. We retrospectively analyzed 4621 cases of papillary carcinoma confirmed by surgery and pathology in Affiliated Hangzhou First People’s Hospital from January 2015 to January 2022. The inclusion criteria were as follows (should have (1) and (2), or (1) and(3)): (1) patients with PTC confirmed by surgery and pathology; (2) pathology after LN dissection confirmed that the LNs in one cervical level were all metastasis-positive or all metastasis-negative; and (3) the FNAC under US-CT fusion navigation confirmed the LN was metastasis-positive. The exclusion criteria were as follows (having one of them requiring exclusion): (1) LN dissection or the FNAC under US-CT fusion navigation was not performed, or the latter confirmed the LNs were metastasis-negative; (2) pathology after LN dissection confirmed that no LN metastasis was detected; (3) pathology after LN dissection confirmed that partial LNs were metastasis-positive; (4) the largest LN diameter < 3 mm; (5) observation of LN affected by artifacts of clavicle or intravenous contrast agent sclerosis; (6) the image could not be observed due to other technical factors; and (7) patients with cervical lymphoma or tuberculosis. Finally, 114 metastasis-positive LNs in 89 cases (28 LNs in 22 cases confirmed by the FNAC under US-CT fusion navigation and 86 LNs in 67 cases confirmed by surgery and pathology) and 143 negative LNs in 114 cases (all confirmed by surgery and pathology) met the inclusion criteria of this study. **Figure 1** shows the characteristics of the study participants in a flow chart.

### 2.2 CT examination

The LNs were scanned with Lightspeed 16 (GE Company, Milwaukee, WI, USA) using the following scanning parameters: 120kV, 250 mA, collimation 0.625 mm × 8, pitch 0.875, and frame rotation time 0.5 s. Patients were positioned supine with scans ranging from the oropharynx to the supraclavicular margin with a slice thickness of 3.75 mm and a slice spacing of 3.75 mm. The contrast agent was iopromide (Bayer Company, Germany) or ioverol (Jiangsu Hengrui Pharmaceuticals Co., Ltd.), with an iodine concentration of 300–350 mgI/mL. The iodine concentration was 300–350 mgI/mL, the injection dose was 60–80 mL, the injection rate was 3–3.5 mL/s, and the patients were scanned 50 s after injection. The average interval between CT examination and surgery was 8 (0–15) days.

### 2.3 Image analysis

**Figure 2:**
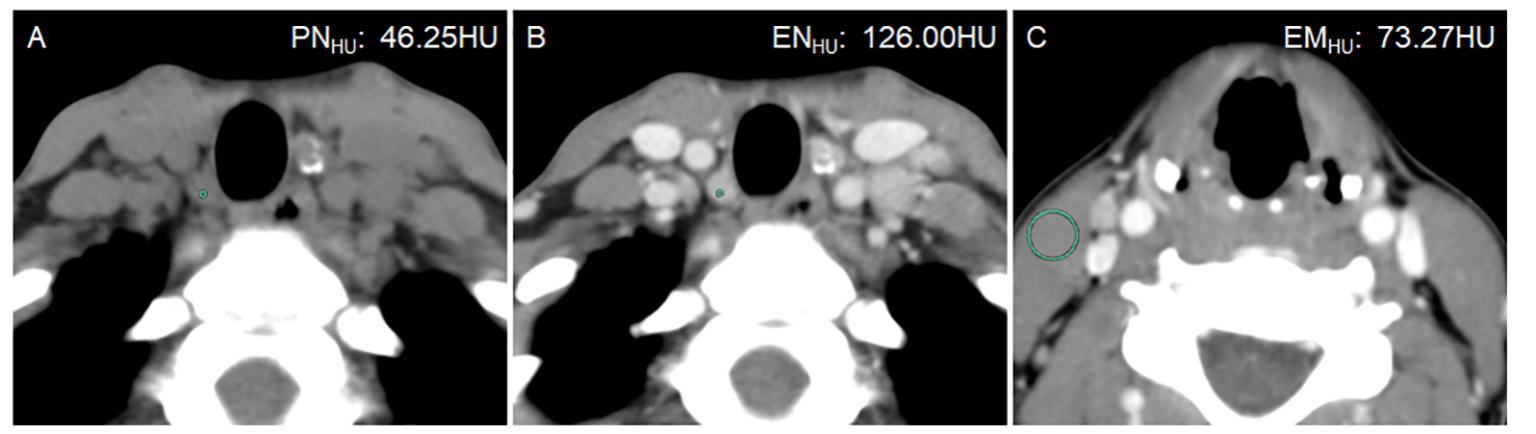
A 21-year-old woman with bilateral PTC. Postoperative pathology confirmed that all lymph nodes in the right level VI were metastasis-positive (5/5). **(A)** PN_HU_ was 46.25 HU. **(B)** EN_HU_ was 126 HU. **(C)** EM_HU_ was 73.27 HU. EN-PN_HU,_ EN/PN_HU_, EN-EM_HU_, and EN/EM_HU_ were 79.75 HU, 2.72, 52.73 HU, and 1.72, respectively.

**Figure 3:**
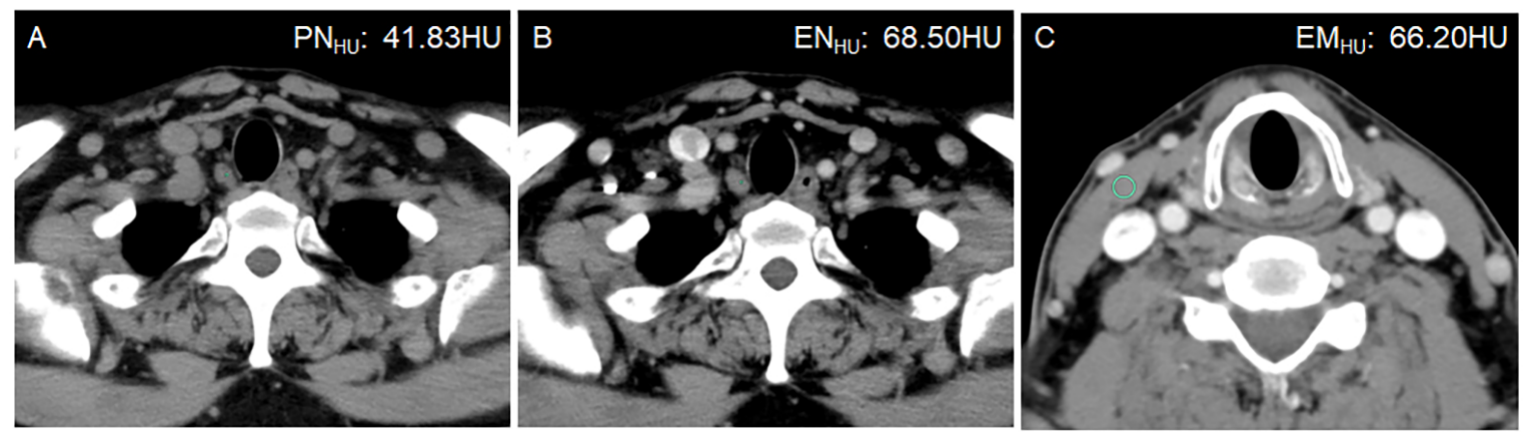
A 50-year-old man with bilateral PTC. Postoperative pathology confirmed that all lymph nodes in the right level VI were metastasis-negative (0/5). **(A)** PN_HU_ was 41.83 HU. **(B)** EN_HU_ was 68.50 HU. **(C)** EM_HU_ was 66.20 HU. EN-PN_HU_, EN/PN_HU_, EN-EM_HU_, and EN/EM_HU_ were 26.67 HU, 1.64, 2.3 HU, and 1.02, respectively.

**Figure 4:**
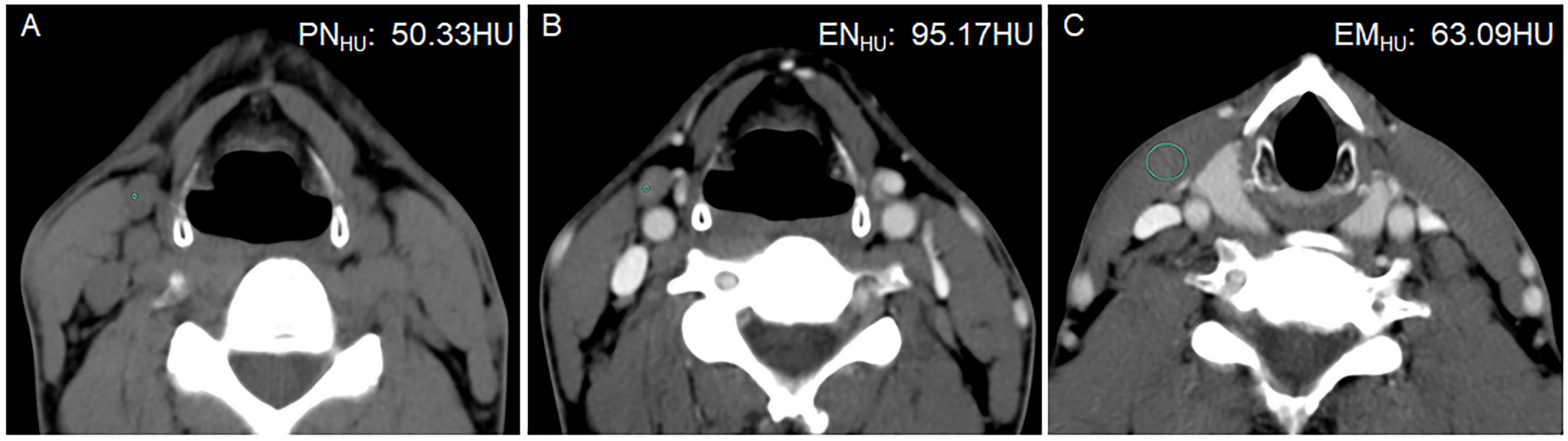
A 42-year-old man with PTC complicated with Hashimoto’s thyroiditis in the left lobe of the thyroid gland. Postoperative pathology confirmed that all lymph nodes in the right level III were metastasis negative. **(A)** PN_HU_ was 50.33 HU. **(B)** EN_HU_ was 95.17 HU. **(C)** EM_HU_ was 63.09 HU. EN-PN_HU,_ EN/PN_HU_, EN-EM_HU_, and EN/EM_HU_ were 44.84 HU, 1.89, 32.08 HU, and 1.51, respectively.

Two head and neck doctors, Han (21 years of work experience) and Zhu (17 years of work experience), separately analyzed the CT data of the picture achieving and communication system (PACS) including the selection of LNs and the measurement of PN_HU_, EN_HU_, and EM_HU_. When selecting LNs, the LN with the largest short diameter was selected. If ≥ 2 LNs had the same short diameter, the LN with the highest enhancement degree was selected to avoid measuring multiple fused or ill-defined LNs. When measuring the Hounsfield units (HU) of LNs, the largest slice of LNs after enhancement was first selected, and the HU of the most obvious enhanced area was measured and recorded. Then, the region of interest (ROI) measurement area of LNs on the plain CT scan was determined according to the three-dimensional positioning technology (**Figures 2**–**4**), avoiding calcification, cystic degeneration, and vascular structures during measurement. The ROIs of LNs were all measured by 3 × 3 pixels. When measuring the CT HU of the sternocleidomastoid muscle, the level of the lower border of the ipsilateral cricoid cartilage was preferred, followed by the level of the glottis, and the thickest region of the muscle was measured using a circular ROI as large as possible. All the parameters were calculated as follows: EN-PN_HU_ =EN_HU_ – PN_HU_, EN/PN_HU_ = EN_HU_/PN_HU_, EN-EM_HU_ = EN_HU_ – EM_HU_, and EN/EM_HU =_ EN_HU_/EM_HU_. Finally, the data from Han were used for statistical analysis.

### 2.4 Statistical analysis

Statistical analyses were performed using R software (version 4.1.0, https://www.r-project.org/) and SPSS software (version 25, IBM Corporation, NY, USA). Continuous data with normal distribution were represented by means and standard deviations. Continuous data with non-normal distribution were represented by medians and quartiles. Classification variables were expressed as a constituent ratio (percentage). The *t* test was used for continuous variables with normal distribution, the Wilcoxon test was used for continuous variables with non-normal distribution, and the *χ* ^2^ test was used for classification variables. The interobserver correlation coefficient (ICC) was used to evaluate the repeatability of the quantitative indicators measured by two doctors, and ICC > 0.75 was considered to indicate the reproducibility of quantitative indicators. The receiver-operating characteristic (ROC) curve of participants was drawn to evaluate the diagnostic performance of quantitative indicators. The evaluation indicators included area under the ROC curve (AUC), sensitivity, specificity, and accuracy. A *P* value < 0.05 indicated a statistically significant difference.

## 3 Results

### 3.1 Distribution of sex, age, Hashimoto thyroiditis, antithyroid peroxidase antibody, and antithyroglobulin antibody levels

The distribution of sex, age, Hashimoto’s thyroiditis, TPO-Ab, and TG-Ab levels in metastasis-positive and metastasis-negative LN groups of PTC is shown in **Table 1**. Age was not statistically different between the two groups (*P* = 0.116). Male patients were more common in the metastasis-positive LN group than female patients (*P* = 0.033). Hashimoto’s thyroiditis was more common in the metastasis-negative LN group (*P* < 0.001). The TPO-Ab (*P* = 0.046) and TG-Ab levels (*P* < 0.001) in the metastasis-negative LN group were higher than in the LN metastasis-positive group.

**Table 1:** Distribution of sex, age, Hashimoto thyroiditis, TPO-Ab, and TG-Ab levels.

	Pathology or cytology		*χ* ^2^/*Z*	*P*
	Metastasis-positive LNs (*n* = 89)	Metastasis-negative LNs (*n* = 114)		
Sex (%)			4.550^a^	0.033
Male	36 (40.4%)	30 (26.3%)		
Female	53 (59.6%)	84 (73.7%)		
Age	39.00 (31.00, 55.00)	45.00 (37.25, 53.00)	1.571^b^	0.116
Hashimoto ‘s thyroiditis (%)			19.447^a^	<0.001
Yes	11 (12.4%)	46 (40.4%)		
No	78 (87.6%)	68 (59.6%)		
TPO-Ab	33.65 (28.00, 47.70)	39.70 (28.00, 727.00)	1.998^b^	0.046
TG-Ab	23.15 (15.00, 32.00)	39.90 (19.80, 144.70)	3.877^b^	<0.001

^a^χ^2^ test; ^b^ Wilcoxon test.

### 3.2 Distribution of LN size, LN division, PN_HU_, EN_HU_, and EM_HU_

The ICC was 0.874–0.994, in which EN_HU_ had the largest CT value, PN_HU_ had the smallest CT value, and EM_HU_ was intermediate (0.951). The distribution of LN size, LN division, PN_HU_, EN_HU_, and EM_HU_ is shown in **Table 2**. No difference in LN size (*P* = 0.975) and EM_HU_ (*P* = 0.561) was found between metastasis-positive and metastasis-negative groups. The incidence of LN metastasis was higher in level VI than in other levels in the metastasis-positive group (*P* < 0.001). PN_HU_, EN_HU_, EN-PN_HU_, EN/PN_HU_, EN-EM_HU_, and EN/EM_HU_ in LN metastasis-positive group were higher than those in the LN metastasis-negative group (*P* < 0.001) (**Figures 2**–**4**). The PN_HU_ of the cases complicated with Hashimoto ‘s thyroiditis (**Figure 4**) and non-complicated with Hashimoto’ s thyroiditis (**Figure 3**) was 44.38 (40.02, 48.00) and 44.00 (39.00, 48.00) (*Z* = 0.371, *P* = 0.711), and EN_HU_ was 76.38 (69.62, 89.86) and 77.50 (64.00, 87.00) (*Z* = 0.999, *P* = 0.322), respectively, in the metastasis-negative group.

**Table 2:** Distribution of LN size, division, and HU.

	Pathology or cytology		*χ* ^2^/*t*/Z	*P*
	Metastasis-positive LNs (*n* = 114)	Metastasis-negative LNs (*n* = 143)		
LN division (%)			29.814^a^	<0.001
Level VI	67 (58.8%)	36 (25.2%)		
Level non-VI	47 (41.2%)	107 (74.8%)		
LN size	5.20 (4.30, 7.00)	5.20 (4.45, 6.60)	0.031^c^	0.975
PN_HU_	48.12 (41.00, 53.94)	44.00 (39.35, 48.00)	3.530^c^	<0.001
EN_HU_	113.39 ± 24.13	77.65 ± 15.93	13.624^b^	<0.001
EM_HU_	63.97 ± 6.14	64.37 ± 4.81	0.582^b^	0.561
EN-PN_HU_	65.84 ± 21.72	34.07 ± 13.63	13.625^b^	<0.001
EN/PN_HU_	2.36 (1.98, 2.75)	1.76 (1.54, 2.02)	9.133^c^	<0.001
EN-EM_HU_	49.42 ± 24.59	13.27 ± 15.41	8.826^b^	<0.001
EN/EM_HU_	1.79 ± 0.40	1.21 ± 0.24	12.160^b^	<0.001

^a^ χ^2^ test. ^b^t test, and ^c^Wilcoxon test.

### 3.3 Diagnostic efficacy of EN_HU_, EN-PN_HU_, EN/PN_HU_, EN-EM_HU_, and EN/EM_HU_ for LN metastasis in PTC

**Figure 5:**
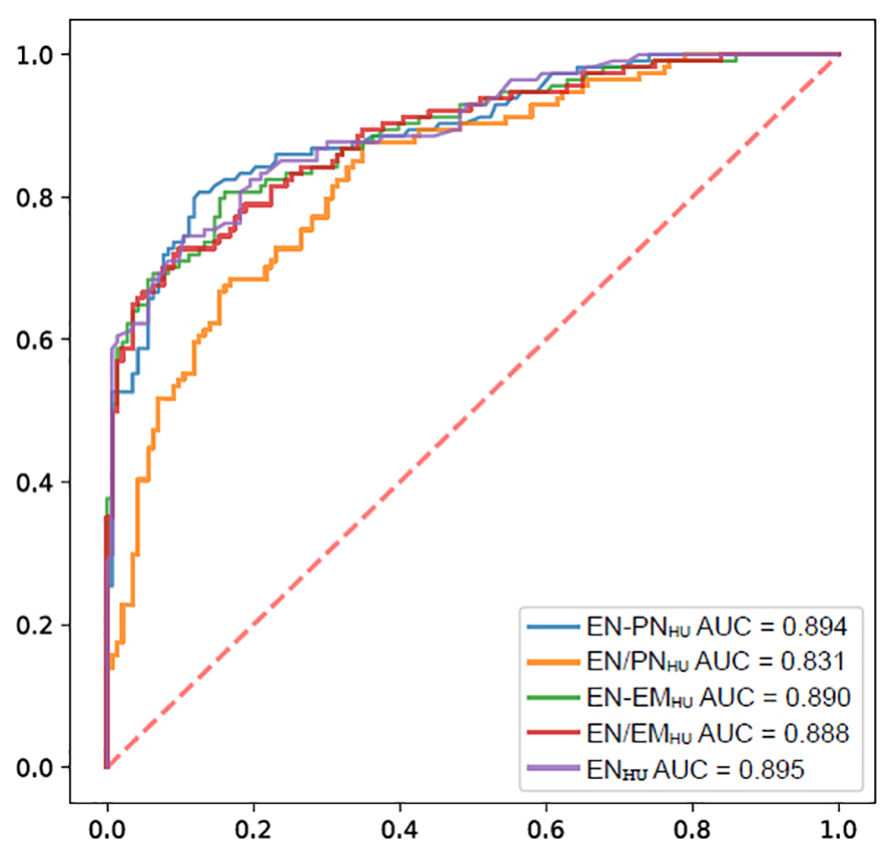
ROC of the EN_HU_, EN-PN_HU_, EN/PN_HU_, EN-EM_HU_, and EN/EM_HU_ for the diagnostic efficacy of LNs metastasis in PTC.

The ROC curves of EN_HU_, EN-PN_HU_, EN/PN_HU_, EN-EM_HU_, and EN/EM_HU_ for differentiating metastasis-positive nodes from metastasis-positive nodes in PTC are shown in **Figure 5**. The AUC and accuracy of EN_HU_, EN/EM_HU_, EN-PN_HU_, and EN-EM_HU_ were highly consistent. EN_HU_ and EN/EM_HU_ had higher specificity, whereas EN-PN_HU_ and EN-EM_HU_ had higher sensitivity. However, EN/PN_HU_ had the highest sensitivity; its AUC, specificity, and accuracy were significantly lower than those of the other four parameters (**Table 3**).

**Table 3:** Diagnostic efficacy of EN_HU_, EN-PN_HU_, EN/PN_HU_, EN-EM_HU_, and EN/EM_HU_ for LN metastasis in PTC.

	AUC	Cutoff value	Sensitivity	Specificity	Accuracy
EN_HU_	0.895	97.3 HU	0.746	0.895	0.829
EN-PN_HU_	0.894	47.8 HU	0.807	0.874	0.844
EN/PN_HU_	0.831	1.9	0.877	0.650	0.751
EN-EM_HU_	0.890	26.4 HU	0.807	0.839	0.825
EN/EM_HU_	0.888	1.5	0.728	0.902	0.825

PN_HU_, plain Hounsfield units of LNs; EN_HU_, enhanced Hounsfield units of LNs; EM_HU_, enhanced Hounsfield units of the sternocleidomastoid muscle; EN-PN_HU_, difference between EN_HU_ and PN_HU_; EN/PN_HU_, ratio of EN_HU_ to PN_HU_; EN-EM_HU_, difference between EN_HU_ and EM_HU_; EN/EM_HU_, ratio of EN_HU_ to EM_HU_; AUC, area under the curve.

### 3.4 Cytological examination guided by US-CT fusion navigation

**Figure 6:**
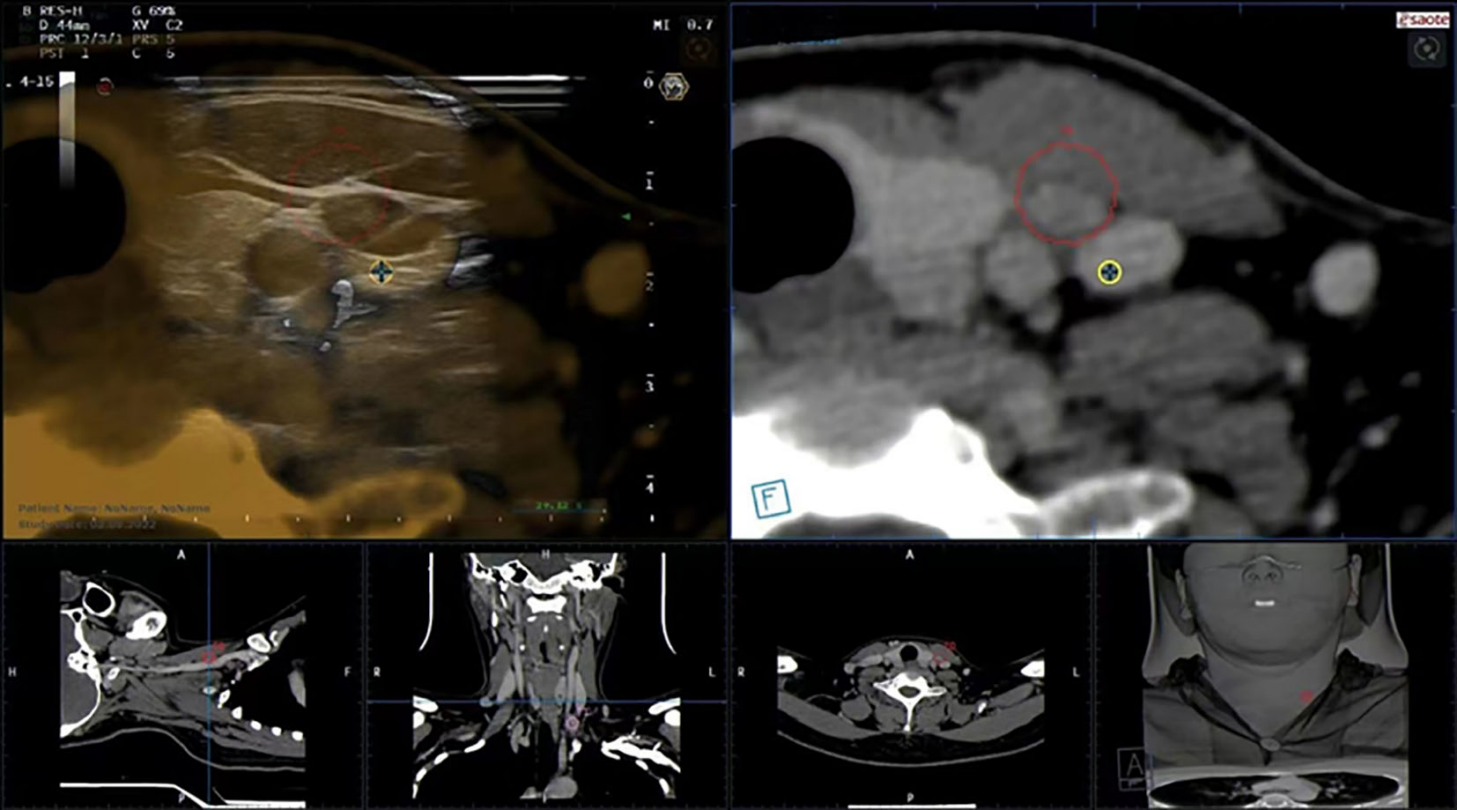
US-CT fusion imaging shows that the LN marked after image fusion was in the red circle, and LN metastasis in PTC was confirmed by FNAC.

In this study, 103 LNs in 73 cases were examined by FNAC with the guidance of US-CT fusion navigation. Finally, 28 LNs in 22 cases were confirmed as metastasis positive. **Figure 6** shows the application of US-CT fusion imaging.

## 4 Discussion

The diagnostic value of CT signs, such as necrosis, cystic degeneration, microcalcification, and significant enhancement in PTC LN metastasis, has been widely recognized (2, 10, 11). Among these signs, cystic degeneration has the highest specificity (96.3%–99.7%) in the judgment of LN metastasis in PTC but the lowest sensitivity (15.1%–24.3%); it was common in patients with extensive LN metastasis (10, 14). The specificity of microcalcification in judging PTC LN metastasis was second only to cystic degeneration, being about 78.5%–96.2%; its sensitivity was equal to or slightly higher than that of cystic degeneration, being about 21.5%–30.0% (10, 14). Significant enhancement refers to a higher degree of LN enhancement than the adjacent sternocleidomastoid muscle (10, 12, 15). Although this standard is simple to use and easy to remember, extensive overlap exists between metastasis-positive and metastasis-negative LNs. In our study, 99.1% (113/114) and 78.3% (112/143) of metastasis-positive and metastasis-negative LNs showed significant enhancement, respectively, which was far from meeting the need for clinical diagnosis. Therefore, quantifying the degree of LN enhancement is important for improving the efficacy of the preoperative diagnosis of LNs.

At present, large differences exist in the delayed CT scanning time of cervical LN metastasis in PTC, such as 25 s, 35 s, 40 s, 45, 50 s, 60 s, 70 s, and 80 s (10–12, 15–18), using one or two phases. It is not difficult to understand that the conclusions drawn may differ using different scanning times; even if the scanning parameters are the same, the results may differ due to individual differences. For example, different conclusions have been drawn by the following scholars with the same delayed 25 s scanning after enhancement. Park et al. (16) analyzed 102 LNs in 42 patients, and the sensitivity and specificity were, respectively, 83%–87% and 93.7%–97.9%, at a cutoff value of EN_HU_ 99 HU. Gürsoy et al. (17) studied 272 LNs in 43 patients, and the results showed that the sensitivity and specificity were, respectively, 93.4% and 99.3%, with the cutoff value of EN_HU_ 109 HU. Su et al. (18) studied 59 LNs in 34 patients and showed that the sensitivity and specificity were 90.62% and 77.78%, respectively, at a cutoff value of EN_HU_ 81.377 HU. The shortcomings of the studies by Park et al. (16) and Gürsoy et al. (17) included the possible deviation of sample representativeness (too many LNs were selected in one patient) and the fact that the LNs did not reach or completely reach the level of comparison between pathology and imaging. The small sample size was the limitation of the study by Su et al. (18). Yoon et al. (10) compared the enhancement degree of lateral cervical LNs and sternocleidomastoid muscle, and the results showed that the diagnostic efficiency was the highest when the difference between them was 35.7 HU, and the sensitivity, specificity, and accuracy were 0.686, 0.788, and 0.730, respectively. However, the limitation of their study was that the LNs did not reach the level of node-to-node contrast between imaging and pathology.

Our medical center adopted a single-phase delayed scan for 50 s to minimize the patient’s radiation exposure, which was similar to the venous scan time used by Yoon et al. (10) and Kim et al. (11), and consistent with that used by Su et al. (18). The following three modifications were made in this study based on the findings of Park et al. (16), Gürsoy et al. (17), Su et al. (18), and Yoon et al. (10), including: (1) the LNs in one cervical level confirmed to be all metastasis-positive or all metastasis-negative, as well as the metastasis-positive LNs confirmed by FNAC under US-CT fusion navigation, were adopted, to ensure that each LN was definitely metastatic or nonmetastatic. Meanwhile, only one LN in the all metastasis-positive or all metastasis-negative level was selected as the study subject so that the sample was more representative (257 LNs were derived from 204 cases); (2) the measurement area of the sternocleidomastoid muscle (the level of the lower edge of the ipsilateral cricoid cartilage) was defined, mainly due to the thicker muscle in this area and uniform density, which were not easily affected by muscle atrophy and partial volume effect; also, the difference between different observers was small (ICC = 0.951); (3) PTC LN metastases were rich in blood supply and showed significant enhancement on CT enhanced images. This study used a small ROI (3 × 3 pixels) was used to measure the most significantly enhanced area. The results reflected the CT HU of LN metastasis to a greater extent, and the difference between different observers was smaller (ICC = 0.994). Our results showed that EN_HU_, EN/EM_HU_, EN-PN_HU_, and EN/PN_HU_ had similar diagnostic efficacy and were significantly higher than those reported by Yoon et al. (10), in which EN_HU_ and EN/EM_HU_ had higher specificity. In contrast, EN-PN_HU_ and EN-EM_HU_ had higher sensitivity. Although the sensitivity of EN/PN_HU_ was higher, the specificityand accuracy were lower and should not be used alone for the judgment of LN metastasis in PTC.

Hashimoto thyroiditis is an autoimmune disease. Positive TPO-Ab was found in 90%–95% of patients and positive TG-Ab was observed in 60%–80% of patients (19–21). In this study, the number of patients in the metastasis-negative group with Hashimoto’s thyroiditis was higher than that in the metastasis-positive group, which might well explain why TPO-Ab and TG-Ab in the metastasis-negative group were higher than those in the metastasis-positive group. Hashimoto’s thyroiditis is often accompanied by cervical LN enlargement, especially in the central group. No reliable report is available on whether Hashimoto’s thyroiditis alters the degree of enhancement of the metastasis-positive or metastasis-negative LNs in PTC. The data of LN metastasis-negative PTC cases were analyzed. The results showed that the plain HU and enhanced HU of LNs in the group with Hashimoto’s thyroiditis were not different from those in the group without Hashimoto’s thyroiditis. Unfortunately, cases of Hashimoto’s thyroiditis were few in the PTC LN metastasis-positive group (only 11 cases). Hence, it was impossible to explore whether Hashimoto’s thyroiditis would change the degree of enhancement of metastasis-positive LNs in PTC.

US-CT fusion navigation is the registration and fusion of US and CT images, which integrates the advantages of high resolution and high detection rate of CT and real-time guidance of US. It has been widely used in the interventional therapy of abdominal organs such as the liver, prostate, and kidney (22), and reported in cervical LN metastasis only by Na et al. (12).

The present study had several limitations. First, most patients with negative LN metastasis had no LNs or small LNs, or enlarged LNs with Hashimoto’s thyroiditis in level VI. Therefore, in this study, the metastasis-negative LNs mostly came from levels non-VI (74.8%). Second, the measured data were obtained from two senior head and neck radiologists. Although the radiologists demonstrated excellent interobserver agreement on PN_HU_, EN_HU_, and EM_HU,_ the interobserver agreement between junior radiologists still needed further confirmation through controlled studies. Finally, our study was a single-center retrospective analysis, thus having some selection bias. Further, prospective multicenter studies should be conducted to validate the findings.

In conclusion, EN_HU_, EN-PN_HU_, EN/EM_HU_, and EN-EM_HU_ had similarly high diagnostic efficacy in the preoperative evaluation of LN metastasis in PTC, with EN_HU_, EN-PN_HU_, and EN/EM_HU_ having higher specificity and EN-PN_HU_ and EN-EM_HU_ having higher sensitivity. Also, they were highly repeatable among different operators and hence worth popularizing and applying.

## Data availability statement

The raw data supporting the conclusions of this article will be made available by the authors, without undue reservation.

## Ethics statement

The studies involving human participants were reviewed and approved by The ethics committee of Affiliated Hangzhou First People’s Hospital. Written informed consent for participation was not required for this study in accordance with the national legislation and the institutional requirements. Written informed consent was not obtained from the individual(s) for the publication of any potentially identifiable images or data included in this article.

## Author contributions

All authors contributed to the study conception and design. Material preparation, data collection were performed by MT and TZ, and statistical analysis by HZ and PW. The first draft of the manuscript was written by JZ and all authors commented on previous versions of the manuscript. The final review and revision was by ZH. All authors read and approved the final manuscript. All authors contributed to the article and approved the submitted version.
